# Rediscovering the wild: MiFoDB brings fermented food microbiomes into focus

**DOI:** 10.1128/msystems.00599-25

**Published:** 2025-08-19

**Authors:** Jacob M. Allen

**Affiliations:** 1 Department of Health and Kinesiology, University of Illinois at Urbana-Champaign 14589, Urbana, Illinois, USA; 2 Division of Nutritional Sciences, University of Illinois at Urbana-Champaign 14589, Urbana, Illinois, USA; University of California Irvine, Irvine, California, USA

**Keywords:** fermented foods, metagenomics, food science, microbes

## Abstract

Fermented foods have sustained human societies for thousands of years, with their microbial communities subtly shaping flavor, nutrient preservation, and health. Yet despite this long-standing relationship, much of the microbial complexity within fermented foods remains unresolved. In recent work, Caffrey et al. (E. B. Caffrey, M. R. Olm, C. I. Kothe, H. C. Wastyk, et al., mSystems 10:e00141-25, 2025, https://doi.org/10.1128/msystems.00141-25) put forth a new tool, MiFoDB, a metagenomic workflow that offers a promising alternative for advancing food microbiome science. By enabling strain-level resolution, functional gene annotation, and microbial tracking across substrates and time, MiFoDB provides a clearer view into the ecological and functional landscape of the fermented food microbiota. This work also bridges gaps between food and human microbiome research and brings us closer to a mechanistic understanding of how fermented foods influence health, helping transform ancient dietary practices into actionable and targeted nutritional strategies for improving human health and well-being.

## COMMENTARY

Fermented foods have been part of the human diet for millennia, developed long before the scientific tools to analyze them. These foods are more than culinary traditions; they are living ecosystems, shaped by human hands but powered by the spontaneous activity of diverse and often unpredictable microbial communities.

Only recently have we begun to uncover just how complex these microbial systems truly are. This growing understanding comes at a timely moment, as fermented foods are being reexamined not only for their cultural and sensory value but also for their potential impact on human health (1). Once staples of everyday diets, fermented foods have largely faded from view as industrialized dietary patterns—characterized by processed, convenience-driven food systems—have become dominant across many societies worldwide. Now, as interest in traditional and microbially rich foods resurges, studies are frequently linking fermented food consumption to reduced inflammation (2–5), enhanced gut barrier function (6) (7), and improved metabolic health (8, 9). These benefits are believed to arise from complex, strain-specific microbial interactions that generate distinct bioactive metabolites and peptides within diverse food ecosystems containing both bacteria and yeast. Yet, until recently, our tools lacked the resolution to fully unravel these complex microbial communities.

Today, advances in microbiome science are beginning to shift that paradigm. Food microbiome studies have historically relied on amplicon-based methods like 16S rRNA or internal transcribed spacer (ITS) sequencing. While useful for broad taxonomic surveys, these approaches typically offer genus-level resolution at best and provide limited insights into their functional potential, or in other words, what the microbes are doing. Metagenomic sequencing, by contrast, enables species- and strain-level resolution and allows for direct analysis of functional genes and pathways (e.g., bioactive metabolite biosynthesis pathways) and opens us up to understanding how these complex communities may operate. In this context, the work by Caffrey et al. (10) is especially timely as MiFoDB provides a much-needed lens to uncover the microbial and functional complexity of fermented foods with greater precision than previously possible.

MiFoDB builds on recent advances by offering a customized alignment-based workflow specifically tailored for fermented food microbiomes. Unlike k-mer-based profilers such as Kraken (11) or clade-specific marker tools like MetaPhlAn (12, 13) that rely on reference databases curated for other ecosystems (e.g., the gut microbiome), MiFoDB supports both mapped and unmapped reads and enables functional gene annotation specific for microbes found in food communities. This is especially valuable for spontaneous “wild” fermented foods like sauerkraut and kimchi, which often harbor novel and understudied microbes. Leveraging a database of over 3,000 genomes, including many newly assembled from fermented food samples, MiFoDB enables researchers to resolve strains, identify novel taxa, and annotate genes involved in key processes such as antibiotic resistance, carbohydrate metabolism, metabolite biosynthesis, and flavor production.

What I think is even more exciting is the potential to track microbial strains across time and food environments and to link those strains to specific functional outcomes. MiFoDB does not simply tell us who is present; it provides a clearer view of what microbes are capable of and why they persist. This opens the door to discovering novel bacterial strains or previously unrecognized metabolic pathways linked to health-promoting or flavor-enhancing compounds. For example, Caffrey and colleagues identify strains associated with histamine—an immune-modulating metabolite—as well as others linked to ethyl maltol, a compound that imparts a caramel-like flavor. These findings suggest new opportunities for designing fermentation workflows that modulate microbial metabolism to enhance the health benefits or sensory qualities of fermented foods.

Beyond pathway discovery, the workflow also enables broader ecological comparisons across different food substrates. This helps identify traits that allow certain microbes to thrive not only in vegetable or dairy matrices but potentially in the human gut. For example, consider fiber degradation. MiFoDB revealed that microbial genes encoding pectate lyases and glycoside hydrolases are enriched in specific vegetable ferments. These enzymes help microbes break down plant polysaccharides, supporting their survival in fibrous food environments such as cabbage. Because different foods contain distinct fiber types, analyses like these provide insight into how microbes adapt to their ecological niches. Such traits may also act as pre-adaptive mechanisms that support colonization and persistence in the gut, where dietary fiber is a key metabolic resource. By connecting these functional insights, MiFoDB encourages us to ask deeper evolutionary and ecological questions: which microbes succeed where, and why?

Overall, MiFoDB is a valuable addition to the growing toolkit for studying fermented foods. Its modular design and open-source accessibility make it adaptable and invite continued refinement by the research community. It complements ongoing work in multi-omics and microbe-host interactions and helps move the field closer to linking fermentation processes to meaningful outcomes, including human health. As interest in fermented foods accelerates across sustainability, culinary innovation, and precision nutrition, robust and customizable workflows like MiFoDB will be essential for building a future where the microbiology of what we eat is as important as its flavor.
